# Contribution of the voluntary sector to mental health crisis care in England: protocol for a multimethod study

**DOI:** 10.1136/bmjopen-2017-019238

**Published:** 2017-11-08

**Authors:** Karen Newbigging, John Mohan, James Rees, Jenny Harlock, Alex Davis

**Affiliations:** 1 Health Services Management Centre, University of Birmingham, Birmingham, UK; 2 Third Sector Research Centre, University of Birmingham, Birmingham, UK; 3 Open University Business School, Milton Keynes, UK; 4 Nuffield Department of Population Health, Oxford University, Oxford, UK; 5 Suresearch, Birmingham, UK

**Keywords:** mental health, adult psychiatry, qualitative research, organisation of health services

## Abstract

**Introduction:**

Timely access to the right kind of support for people experiencing a mental health crisis can be problematic. The voluntary sector (VS) plays a key role in providing support and enabling access, but there is a knowledge gap concerning its contribution and interface with public services in mental health crisis care. This study aims to address this.

**Methods and analysis:**

The study has three empirical elements: (1) a national survey of voluntary sector organisations (VSOs) in England and national stakeholder interviews to develop a typology of organisations and interventions provided by VSOs; (2) detailed mapping of VS services in two regions through interviews and extending the national survey; (3) four case studies, identified from the regional mapping, of VS mental health crisis services and their interface with National Health Service (NHS) and local authority services, at both a system and individual level. Data collection will involve interviews with commissioners; VSO and NHS or local authority providers; and focus groups with people who have experience of VSO crisis support, both service users and carers; and mapping the crisis trajectory of 10 service users in each study site through narrative interviews with service users and informal carers to understand the experience of VSO crisis care and its impact.

**Ethics and dissemination:**

The University of Birmingham Humanities and Social Sciences Ethical Review Committee granted ethical approval (reference ERN_16–1183) for the national and regional elements of the study. Ethical review by the Health Research Authority will be required for the case study research once the sites have been identified from the first two elements of the study. A range of methods including a policy seminar, publication in academic journals and a tool kit for commissioners and practitioners will be produced to maximise the impact of the findings on policy and practice.

**Strengths and limitations of this study**

- This study addresses a key knowledge gap in relation to the contribution of the voluntary sector to mental health crisis care and to theoretical understandings of the third sector and relationship with the public sector.
- The multimethod design combining quantitative and qualitative methods enables a comprehensive investigation of the breadth of the voluntary sector contribution, the interface with public sector services and the pathways of people in a mental health crisis.
- People with lived experience of a mental health crisis are involved in all aspects of the study, recognising the epistemological importance of their contribution to conceptualising crisis, identifying preferences and defining positive outcomes for mental health crisis care.
- Clinical outcomes and comparisons with different types of service provision are beyond the scope of this study, but it provides a platform for further research of this nature.

## Introduction

If not managed well, the experience of a mental health crisis can have a long-lasting and negative impact and may influence the capacity for self-management and willingness to seek help in the future. Consequently, the provision of effective mental health crisis support has been a cause for concern since the replacement of institutional care with community-based services. In an English context, these concerns still prevail and mental health crisis provision remains both inconsistent and inadequate with many people in crisis unable to access the help they need, when they need it and being dissatisfied when they do.^1 2^

While statutory services form a key strand of the mental health crisis response, the voluntary sector (VS), also commonly described as the third sector (and non-profit or non-governmental organisations in other jurisdictions), has played a key role in response to failures of statutory provision, filling gaps in service provision or in responding to policy initiatives.^3^ The VS has developed innovative models of care: valuing accessibility, self-organisation, user-defined conceptions of crisis, informality and relational-based approaches in contrast to the often inflexible, risk averse and biomedical approach of statutory services. It has played a particularly important role in meeting the needs of specific communities, notably for people from Black, Asian and minority ethnic (BAME) communities who may avoid using statutory services because of fear and concerns relating to a lack of understanding of cultural heritage, racist treatment and limited range of options offered by statutory services.^4^

VS crisis support ranges from helplines to peer support to crisis houses and alternatives to inpatient care, and VS providers may be large national charities delivering a range of responses in different localities or smaller local social enterprises providing a specific service, such as a crisis café. However, the range of provision in the VS is not well understood nor how such services can effectively interface with those provided by statutory services, despite this being promoted by current policy.^5^ This gap in knowledge needs addressing to support policy and practice development that builds on an understanding of and demand from service users for a plurality of provision and different approaches to mental health crisis care. The current economic situation of the National Health Service (NHS) and local authorities also underlines the obligation on public services to use their resources to best effect. It is, therefore, extremely timely to address this knowledge gap and to identify the policy and practice implications.

### Background and rationale

#### Conceptions of a mental health crisis

The term ‘crisis’ in a mental health context covers a broad range of needs, including an individual’s capacity to cope, available resources that can be mobilised and the effectiveness of ongoing care and support.^6^ The definitions of crisis currently in use distinguish a pragmatic service-oriented approach (ie, a person coming to the attention of crisis services because of a serious relapse of an existing mental health condition), self-definitions of crisis, risk-focused definitions and negotiated definitions (ie, negotiated collaboratively between service users, carers and professionals).^7^ This study begins with a broad, and thus inclusive, conception of a crisis as a ‘turning point’, such that a mental health crisis is personally disruptive but can provide opportunities to strengthen personal and social resources and to anticipate and manage mental health, leading to improvements in health and well-being.^8^

### Role of the VS in mental health crisis care

The range and potential complexity of mental health crises means that there is not a single crisis care pathway and the configuration of crisis care pathways varies depending on when and how someone first presents with a crisis.^5^ The configuration of these pathways is better understood where the first approach is to statutory services (eg, general practitioner (GP), NHS crisis services or accident and emergency), but there will also be people who are reluctant to use statutory services as their first port of call and may turn to the VS in a crisis.

The VS has been conceptualised as a ‘third’ space of organisations between the state and market, comprising charities, social enterprises and community and grassroots groups, which exhibit a set of usually linked attributes, notably the existence of an explicit social mission, closeness to communities and beneficiary involvement in governance.^9 10^ This is underpinned by a sector ethos that typically values accessibility, self-organisation, service-user-defined outcomes, informality and relational-based approaches.^11^ It is argued that this relational aspect provides a ‘comparative advantage’ in terms of addressing the needs of particular disadvantaged groups,^12^ and thus, voluntary sector organisations (VSOs) may occupy a specialist ‘niche’ within a wider ecosystem of mental health crisis support. The sector has provided leadership in terms of developing recovery-oriented approaches and peer support,^13 14^ developed innovative models of care^15^ and, offer an alternative, and potentially complementary, adjunct to statutory crisis provision through providing a non-medical response that focuses on the person’s situation and seeks to empower them in dealing with their crisis.

The Crisis Care Concordat, introduced in 2014,^5^ has stimulated the development of a range of VS initiatives, and the importance of different agencies working together to ensure that there is an effective crisis care pathway is repeatedly emphasised in policy^15 16^ and service evaluation.^17^ Where VSOs work most closely in ‘partnership’ with the public sector, the relationship has been theorised as a collaborative one arising from the inherent limitations of the two sectors in providing collective services.^18^ Gajda’s^19^ theoretical framework and Morrissey *et al*’s work on the mental health system^20 21^ provide a basis for exploring the extent of collaboration between VSOs and the public sector and how effectively the voluntary and statutory sectors are working together to deliver mental health crisis care, both at a system and individual level.

### Existing evidence base

The research endeavour has largely focused on statutory provision, notably in relation to Crisis Resolution Teams,^22–24^ and there are key gaps in the evidence base for effective mental health crisis care, notably in relation to VS provision.^7^ Crisis houses, usually provided by VSOs, are currently being developed, and there is a growing body of evidence that service users prefer residential crisis houses to inpatient psychiatric care and they are less stigmatising and coercive, thus proving a viable alternative for people not needing close supervision and observation.^25 26^ In addition, there is some evidence that they may be more cost-effective^27 28^ and, in building on informal peer support, extend the networks and repertoire in the event of future difficulties.^29^

While large national charities tend to have a high profile in this field, less is known about the range of services provided by smaller organisations, particularly those operating informally, or about how the division of labour between providers operates on the ground. In addition, variation in the distribution of VS services and the extent to which their contributions are integrated into local crisis care pathways is not clear. A mismatch between patterns of social need and VS provision has been identified, such that access to effective crisis support that includes a range of VS provision is likely to be something of a ‘postcode lottery’.^30^

## Study aim and objectives

The primary aim is to identify the range and nature of the VS contribution to effective crisis care pathways in mental health in order to strengthen the crisis care response. The study has five key objectives:

1. To identify the different types of VS support being commissioned and provided to respond to the care needs of people experiencing a mental health crisis.
2. To develop a taxonomy of the different organisational types (eg, social enterprise, charity, Community Interest Company, etc) and forms (eg, national, local, etc) of VS support available, service models including characterisation of their relationships with statutory provision and populations served.
3. To investigate the experience of different stakeholders and outcomes for service users of the contribution of different types of VS support to the crisis care system.
4. To identify the factors and processes that facilitate the successful contribution of the VS to effective crisis care pathways.
5. To identify the policy and practice recommendations to strengthen the mental health crisis care response, including how best the VS and statutory sector can work together to ensure a rapid and appropriate response.

The scope is mental health crisis care in England.

## Research design and methods

The study is designed to work from national level perspectives and data on the contribution of the VS, through to the experience of individual service users and informal carers of local VS services. The balance of qualitative and quantitative material varies at these different levels of investigation with the quantitative material being relied on for what Sayer^31 32^ characterises as an ‘extensive’ approach, describing broad patterns of a phenomenon, while the qualitative material is drawn upon for ‘intensive’ investigations of outcomes and processes. Case studies are particularly useful for enabling a real-time exploration of phenomena that are complex and dynamic,^33^ and a case study design, therefore, involves an intensive examination of the VS contribution and has the potential to contribute to theoretical insights that can then be applied in other contexts. The study design ensures that the qualitative work is always capable of being related to the wider picture through locating the qualitative data in a typology of VSOs derived from the quantitative data.

**Figure 1:**
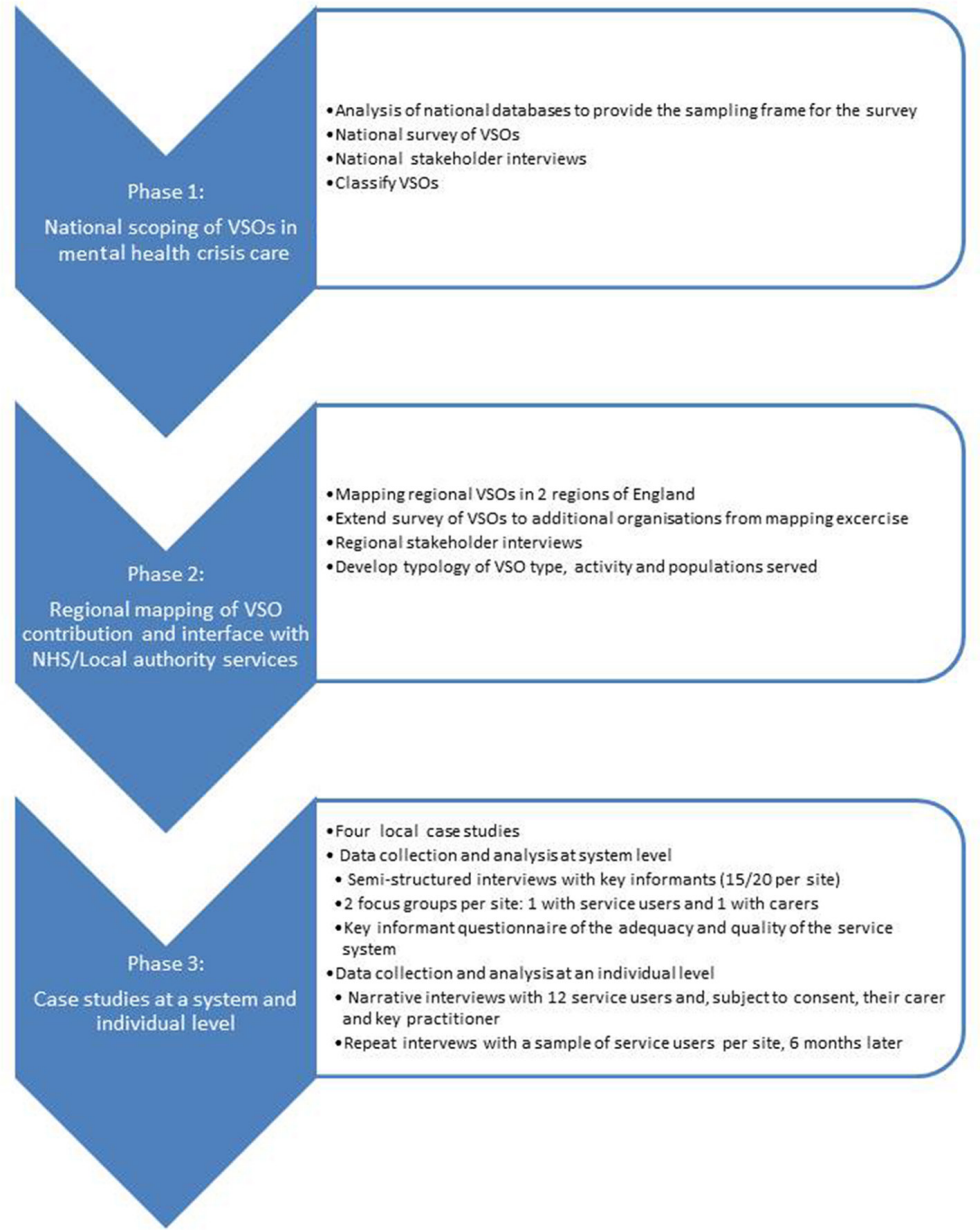
Research design. NHS, National Health Service; VSO, voluntary sector organisation.

The research design involves the use of multiple methods to provide a comprehensive and detailed analysis of the contribution of the VS to mental health crisis care, undertaken in three phases (see figure 1). The quantitative and qualitative packages complement one another, with phase 1 providing a detailed description of the types of organisations involved in mental health crisis care delivery, the details of the types of services they provide and populations served. This macrolevel picture will be developed through the following two phases of the study, providing an in-depth investigation of VS provision, with emphasis on qualitative methods and a focus on process, system-level working and individual experience.

### Phase 1: National scoping exercise

The methods for building a national picture of the contribution of the range of VS providers of crisis care are: (A) a national survey of VSOs to identify the type of crisis support being commissioned and provided and to whom, the type of organisation providing the support and main methods of working and (B) a selected sample of interviews with national stakeholders (Department of Health, NHS England, professional and service user organisations) and national VSOs to provide further detail on the different forms of VSOs and the type of crisis support they provide and how this contributes to the crisis care pathway.

The VS in the UK contains over 200 000 registered and regulated non-profit organisations taking various legal forms. Building on the work of the Third Sector Resource Centre (TSRC), three data sources will be used to ensure that the sampling frame for survey and case study research is as robust as it can be:

1. The Crisis Care Concordat database and websearching to identify the main VSOs providing mental health crisis support.
2. The Register of Charities, developed by TSRC, covering approximately 160 000 registered charities in England and Wales, with detail on their finances and activities for a 20-year period and classified organisations according to subsets of the ICNPO (International Classification of Nonprofit Organisations) classification.^34^
3. Procurement data, from Clinical Commissioning Groups (CCGs) and local authorities (LAs) about transactions with external parties of a value in excess of £25 000.

#### A. National survey of providers

**Figure 2:**
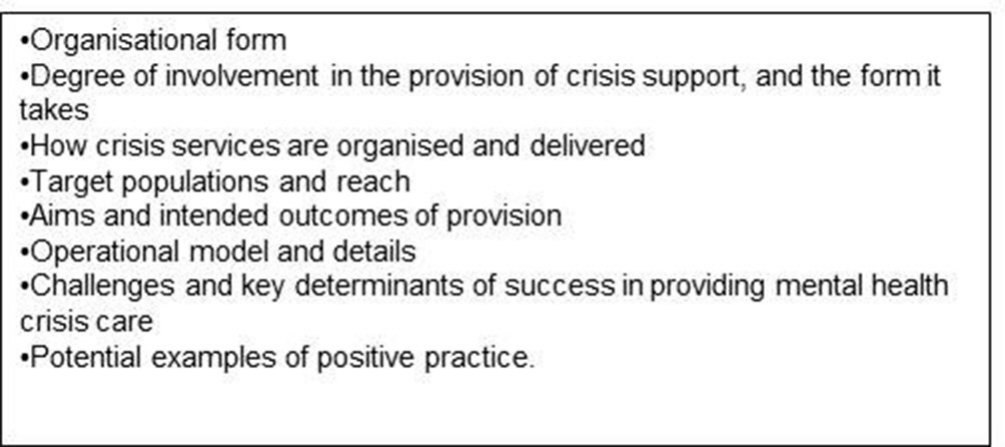
Themes covered by the national e-survey.

A structured electronic survey of VS providers, using Bristol Online,^35^ to the VSOs identified from the database analysis, covering the themes in (figure 2), will combine tick boxes and opportunities to provide free-text responses.

The survey tool will be piloted with a small number of VSOs (n=6) before wider distribution. Response rates will be monitored and telephone follow-up will be employed to encourage response.

Quantitative data from the questionnaires will be used to generate descriptive statistics on the characteristics of the organisations providing services, their activities, resources and distribution. Qualitative free-text responses will be imported into NVivo for analysis and categorised into emergent themes and reported alongside the quantitative data. A typology will be developed to map the different organisational forms, populations served, interventions of VSOs in mental health crisis care and their relationship with public services.

#### B. National stakeholder interviews

Interviews (n=20–25) will be undertaken with a purposive sample of national stakeholders recruited from policy-makers; professional bodies; VS providers; and service user and carer organisations. The interviews will explore:

- The nature of the contribution VSOs can make to mental health crisis care;
- Effective ways of integrating this with statutory services;
- Challenges and key determinants of success in providing mental health crisis care;
- Potential examples of positive practice;
- The future for mental health crisis care.

### Phase 2: Regional mapping

Phase 2 builds on the national scoping exercise to elicit detail on the different types of VSO provision in a regional context. There are three objectives in doing this: (1) to ensure that VSOs that do not describe themselves as crisis providers but nevertheless have this within their provision are included within the study; (2) to investigate the interface with statutory provision and (3) to identify potential variations in access and the role of VSOs, if any, in addressing these within each region.

Two regions will be identified and chosen to reflect demographic diversity likely to be associated with varying levels of need for crisis support and will include large metropolitan contexts with variation in deprivation and demographic diversity, rural/semirural communities with relatively stable and prosperous populations and coastal towns with deprivation and a more transient population. There will be two main data collection methods: (1) targeted interviews with commissioners and providers (n=10 per region) to identity additional activity that has not been picked up through the national scoping and explore the regional context for crisis care; the interface between VSOs and statutory services and what factors facilitate effective crisis care pathways and (2) extending the e-survey used in phase 1 to organisations identified through these interviews.

This will generate further quantitative data, on the activities and resources of regional and local organisations not evident in the national quantitative mappings, and more detail on the roles, resources and relationships of these organisations, of a qualitative kind being drawn from interviews and documentary sources including:

- What voluntary organisations are actually providing;
- Management and funding arrangements;
- How they interface with public sector (and for-profit) agencies, particularly the principal mental health trusts, and how care pathways work in the different regions;
- The experiences of VS provision from different perspectives;
- The role of commissioning agencies;
- Assessment of the quality of services;
- Capacity issues including the availability of financial and human resources.

The resultant data will be analysed to provide an analysis of variation within and between the two regions, the factors that have shaped this and the potential impact on crisis care delivery at a local level. This detailed regional analysis will refine and add to the initial taxonomy developed in the first phase, which will be used as a sampling frame to guide the selection of case study sites for detailed data collection at a local level.

### Phase 3: Case studies of the VS contribution

This is the heart of the study and provides an in-depth understanding of the contribution of the VS within the overall crisis care system and at the individual level of service users and carers.

Four contrasting case study sites will be identified from the output of phases 1 and 2, using two key sampling criteria.

- Geography: case study sites will be selected to include VS provision in both rural and urban settings in England and include cases with a highly diverse population from BAME communities.
- Types of VS provision: cases will be selected to contrast in terms of the types of VS provision identified from the first two phases, for example, a case with a crisis house and one without, and size (eg, local vs national).

A realist approach to sampling will be adopted, recognising that case study research moves back and forward between ‘ideas’ and ‘evidence’ and is consequently iterative.^36^ Within each case study, data will be collected to understand how the crisis care system operates as a whole and at the individual level for people experiencing a mental health crisis.

### System level analysis

**Figure 3:**
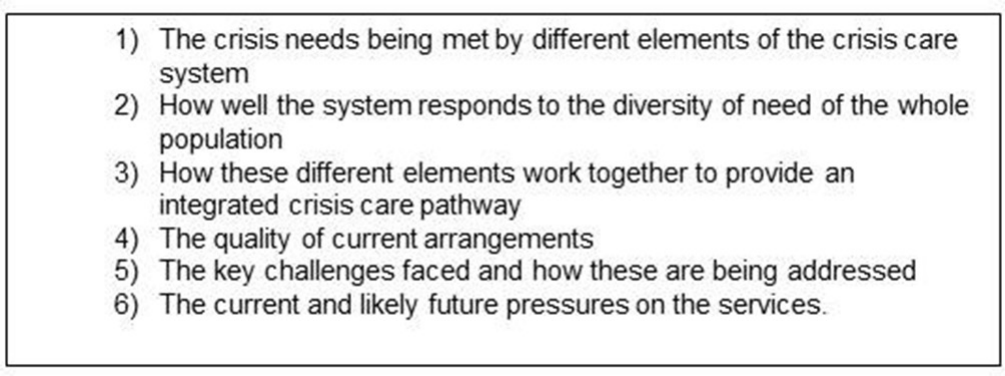
Topic guide for stakeholder interviews in phase 3.

The focus for this is to identify how the different organisations providing crisis care are working together at a local level and what factors facilitate effective integration so that services and their carers experience an easy journey to accessing appropriate support. This will include investigating the extent to which small-scale community groups also assist in the provision of crisis support. In each case study site, three methods will be used:

1. Semistructured interviews with key stakeholders (n=15–20): commissioners; VS providers; NHS/LA managers; mental health practitioners providing mental health crisis support (hospital and community based) and front-line staff, notably the police and GPs. Participants will be identified and recruited from information collected in phase 2. The interview topic guide is provided in figure 3.
2. Two focus groups will be held, one for service users and one for carers to understand experiences of VS provision and how this fits within the crisis care system. The focus groups provide an opportunity for a ‘collective conversation’^37^ with service users and carers as to their experience of crises and the crisis care pathway. This data will provide an important reference point for how these meanings are enacted in the response of VSOs and the wider system.
  Participants will be recruited via the VSOs, service user and carer organisations, LAs and NHS Mental Health Trusts in each case study site. Steps will be taken to ensure diversity in the sample in terms of demographic characteristics, range of mental health problems and crisis experience. All participants will be invited to complete a brief questionnaire at the end of the focus group to capture brief demographic details. The criteria for inclusion are:

- Experience of using VS crisis care within the past 2 years
- Age ≥16 years
- Capacity to be involved in a research interview.

Interpreters and signers will be recruited, as appropriate, to ensure that the participants are a diverse sample. The lines of inquiry for the focus groups will cover:

- Experiences of the crisis system
- How this experience contributed to recovery
- What worked well
- What needs attention
- Differences in experience between engagement with the voluntary and statutory sectors.

1.  A survey of local views of key informants, identified from the regional mapping in phase 2 and stakeholder interviews, using a questionnaire to provide an assessment of how well the crisis system is working. The questionnaire will be developed from the work in a US context by Morrissey *et al*^20^ and informed by a literature review of measures of system integration. It will provide data on the different stakeholder perspectives on the quality and the adequacy of the local crisis system, with questionnaire items evaluating both individual organisations and the system of organisations. This broad systems evaluation will provide a useful adjunct to the interview data.^21^ The questionnaire data will be analysed using descriptive statistics and the qualitative data analysed, as previously, to identify the main themes influencing how the VS is contributing to crisis care at a local level.

### Individual level analysis

The focus of this element of the case studies is to map crisis care trajectories for individuals to develop a granular picture of this journey and how the interface between the person experiencing a crisis and different services is shaped. Participants will be recruited via VSOs, LAs and NHS crisis services and service user organisations. The criteria for inclusion are:

- Experience of using VSOs and an episode of NHS care within the last 12 months to support them with crisis management
- Age ≥16 years
- Capacity to be involved in a research interview.

There will be 12 service user participants per site (n=48) and, subject to their consent, a carer or family member and practitioner. Not all service users will have a family member or carer or consent to them being interviewed and practitioners may well relate to more than one service user and so, the anticipated total number of service user/carer/practitioner interviews will be in the region of 60–80 across the four sites. For each person, information on the crisis care trajectory will be gathered through separate narrative interviews with the person, their carer (if appropriate) and the VS practitioner.^38^ The narrative method will focus on encouraging participants to tell stories about their experiences of and engagement with current support services for mental health crises, including:

- The nature of the mental health crisis
- Which services were used, when and what for
- Access to these services and factors influencing this
- Experience of these different services
- The difference using these services made to individual capacity to deal with the crisis
- What the person would do in the event of a mental health crisis in the future.

Participants will also be invited to complete a questionnaire to provide demographic details, as with the focus group participants. A purposive sample of six service users per site will be followed up at a 6- 9 month interval to gather further reflections on their experience and draw comparisons with their original narrative. Interview data will be recorded and transcribed using NVivo V.11 to assist in data management and analysis. Data analysis will involve comparing their different accounts of the mental health crisis and building a process map of the crisis care trajectory, including how the interface between VSOs and statutory services was negotiated.

## Data analysis

The analytic strategies reflect the research objectives to understand the contribution of VSOs in responding to people experiencing mental health crises.

1. Classification of the organisations and activities undertaken by VSOs to develop a taxonomy of the range of contributions and use as a sampling frame for selection of the case study sites.
2. A thematic analysis of national stakeholder interviews to identify additional VSOs and refine the taxonomy.
3. Content analysis of documents and commissioning strategies to identify the VSO contribution and levels of investment.
4. Analysis of interview and focus group data using the Framework method^39^ to identify key themes and investigate relationships between different themes and different types of participant.
5. Exemplar care pathways will be mapped to provide a detailed understanding of the interface between VSOs and NHS, LA and other public sector services.

All interviews will be recorded and transcribed and NVivo V.11 used to manage and code the data and to compare data across different participants, case study sites and types of VSOs.

Data synthesis will be an iterative process focused on the research objectives and exploring the relationships and tensions between the following variables:

- The type of VS provision and activities
- The type of crisis needs
- Individual respondent characteristics and methods
- Organisational form and commissioning arrangements.

The analysis will, therefore, locate the qualitative data on experiences and outcomes within the framework provided by the quantitative data and will be integrated in the reporting of the findings.

## Ethical considerations

### Approval by ethics committees

Standards of good practice for research will be followed,^40^ and the project will be undertaken in compliance with the Data Protection Act. This study has received ethical approval from the University of Birmingham Humanities and Social Sciences Ethical Review Committee for the first two phases (reference ERN_16–1183). The study will require Heath Research Authority approval for phase 3 once the case study sites have been identified, as a consequence of this earlier work. The main ethical issues that arise from this research are achieving voluntary informed consent, confidentiality, anonymity and privacy and discussion of distressing or upsetting topics.

#### A. Consent

Written consent to participate will be obtained from all participants in focus groups and interviews prior to data collection. Capacity to participate in the study will be assumed unless a potential service user participant clearly demonstrates that they lack the capacity to do so, using a range of methods to establish this^41^ reflecting current legislation and good practice.

#### B. Confidentiality and data protection

Participants will be advised that what they say will be treated as confidential, unless they reveal potential harm to themselves or someone else. Care will be taken to maintain the confidentiality of records and prevent disclosure of identities of research participants, in accordance with accepted codes of conduct and the Data Protection Act.

#### C. Risks and hazards

An agreement about the arrangements that need to be in place to manage any adverse reaction will be made with the organisations at each of the sites before focus groups or interviews take place. In the event of anyone becoming distressed, the interview or focus group will be brought to a close and only restarted if or when the individual concerned feels ready.

## Patient, service user and public involvement

The study draws on a range of direct experiences of crisis support and different interpretive frameworks, by ensuring that service users, carers and members of the public are active participants in the research process, able to shape, change and challenge the research process and the knowledge development.^42^ There will be service user involvement in all aspects of this study, via Suresearch, a network of over 100 mental health service users, survivors and their allies involved in research and education.^43^ Cognisant of good practice,^44 45^ service users, carers and members of the public will be actively involved as follows:

(A) As coresearchers: five people with lived experience of a mental health crisis will be recruited as coresearchers, via Suresearch, and involved at all stages of the research. Each coresearcher will be buddied with an academic for ongoing support and supervision and training will be provided, as necessary, and cover:

- Ethical considerations and good practice in research
- Designing research questions
- Collecting data through interviews and focus groups
- Analysing data
- Reporting findings, including effective dissemination for impact.

(B) Members of a Service User Reference Group: to act as a critical friend, to inform the development of the research tools and lines of inquiry and comment on emergent findings. This group will consist of eight people with representation from the public, carers and mental health service users, and specific attention will be paid to ensuring that diverse groups are represented.

(C) Representation on the Study Steering Group: members of this Service User Reference Group will be represented on the Project Steering Group, which will provide overall governance of the project and ensure that the key milestones are met as well as providing expert advice.

The National Involvement Partnership’s National Involvement Standards (National Survivor User Network for Mental Health)^46^ will be used as a framework for reflecting on the quality of involvement through the course of the study.

## Dissemination

The project will generate knowledge to respond to the national and local priority to improve access to mental health crisis care. Local launch events in case study sites will enable reflective discussion about the implications of project findings for stakeholders, including commissioners, practitioners, VSOs, service user and carer organisations, and a national seminar, will maximise impact on policy-making. Findings will be given national and international resonance through academic publications and practitioner publications (including a practical guide for commissioners and practitioners).

## Conclusion

The research design and use of multiple methods, combining quantitative and qualitative approaches, enables a comprehensive investigation of the range of VS mental health crisis services, their interface with public sector services and the care pathways of people in a mental health crisis. The study findings will improve our understanding of the contribution of the VS to mental health crisis support in England and will have the potential to inform the development of beneficial interventions and services to improve the management of mental health crises in England, the wider UK and elsewhere.

## Supplementary Material

Reviewer comments
