# Supplementary material for: Contribution of the voluntary sector to mental health crisis care in England: protocol for a multimethod study
Source: BMJ Open. 2017 Nov 8;7(11):e019238. doi: 10.1136/bmjopen-2017-019238 (PMC5695387; doi:10.1136/bmjopen-2017-019238)
Supplement: Reviewer comments [file bmjopen-2017-019238.reviewer_comments.pdf]

PEER REVIEW HISTORY

BMJ Open publishes all reviews undertaken for accepted manuscripts. Reviewers are asked to complete a checklist review form (**http://bmjopen.bmj.com/site/about/resources/checklist.pdf**) and are provided with free text boxes to elaborate on their assessment. These free text comments are reproduced below.

ARTICLE DETAILS

**TITLE (PROVISIONAL)**	The contribution of the voluntary sector to mental health crisis care in England: a study protocol for a multi-method study
**AUTHORS**	Newbigging, Karen; Mohan, John; Rees, James; Harlock, Jenny; Davis, Alex

This article was not externally reviewed at BMJ Open. Protocols that have been independently assessed prior to submission to BMJ Open are usually fast-tracked to publication on the grounds that further substantial changes will not be possible. This independent assessment will usually be external, independent review for both a substantial grant award from a non-commercial or government funder and ethics approval.
